# Correction to “Capturing the Elusive Curve-Crossing in Low-Lying States of Butadiene With Dressed TDDFT”

**DOI:** 10.1021/acs.jpclett.6c00984

**Published:** 2026-04-30

**Authors:** Davood B. Dar, Neepa T. Maitra

We report three errors in the implementation of the DTDDFT method in the original paper First, the DTDDFT results (both DTDDFT_a_ and DTDDFT_s_) were computed with a factor of one-half applied to the dressing term *X* in eqs 16 and 17 due to a bug in the code. Second, the DTDDFT_a_ result in Figure 1 used Kohn–Sham single-excitation frequencies in the numerator of eq 19, in place of the adiabatic frequencies. While this would be a valid approximation for a possible DTDDFT variant, it is not consistent with the variant presented in eq 19; likewise the results with the one-half factor could be considered as another DTDDFT variant. The DTDA results and the discussion about them remain consistent with the formulas presented in the Supporting Information. Lastly, the DTDDFT (DTDDFT_a_ and DTDDFT_s_) calculations were not fully iterated to self-consistency.

**Figure 1:**
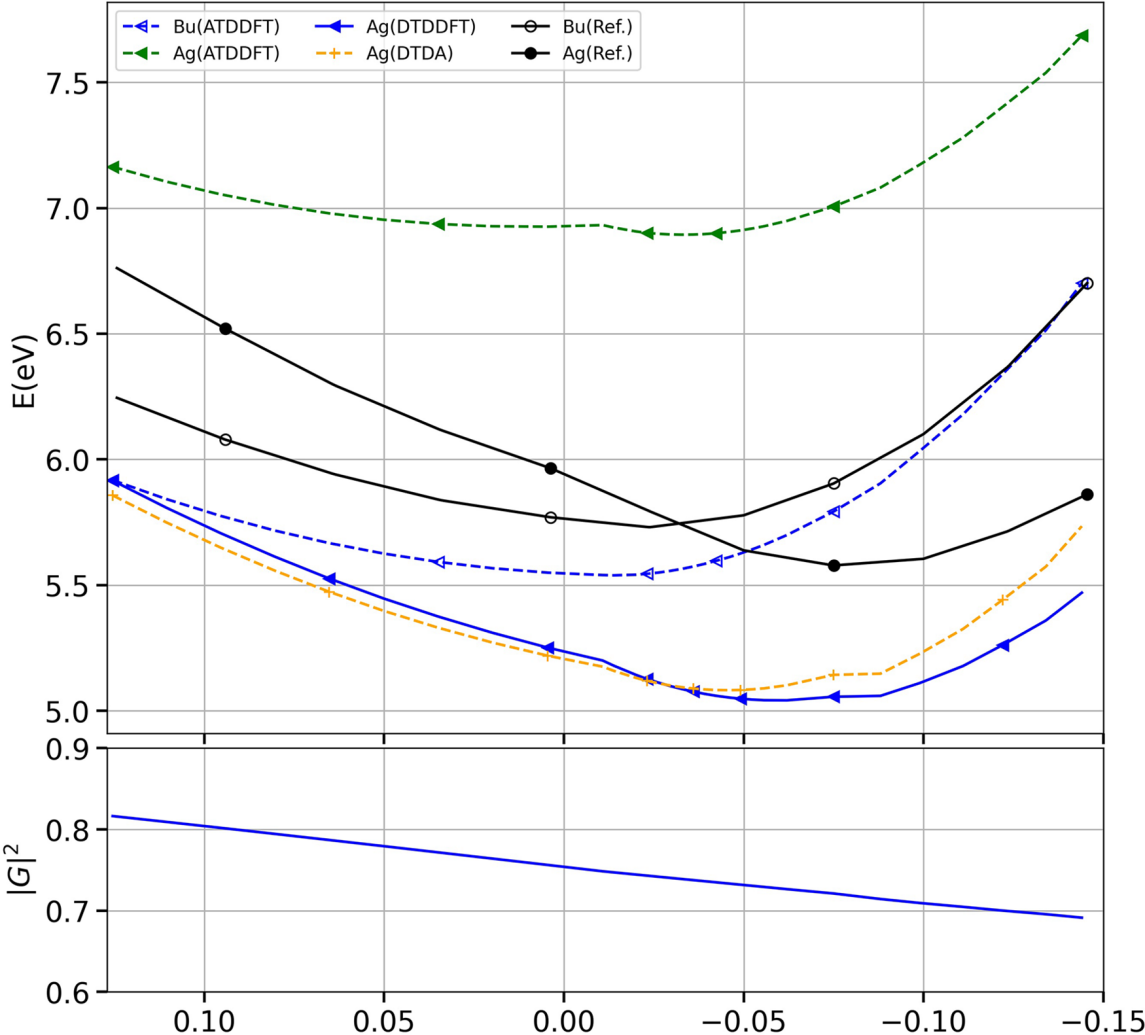
DTDDFT_a_ results computed in accordance with the equations presented in the main paper and corresponding to Figure 1, using the PBE0 functional and the cc-pVTZ basis set.

These errors impact the following plots:

- Figure 1 (main text): The Ag­(DTDDFT_a_) curve and the *G*_1_^2^ value in the lower panel have been computed with the three errors above, while the remaining curves in Figure 1 (adiabatic TDDFT and DTDA) are correct. The figure with the corrected calculation is shown below (see also discussion below).
- Figure 2 (main text): The Ag (DTDDFT_a_ and DTDDFT_s_) curves have been computed with the errors above.
- Figure 1 (Supporting Information): The Ag­(DTDDFT_a_) curves have been computed with the three errors above. (See Figure 3 and discussion below)

**2:**
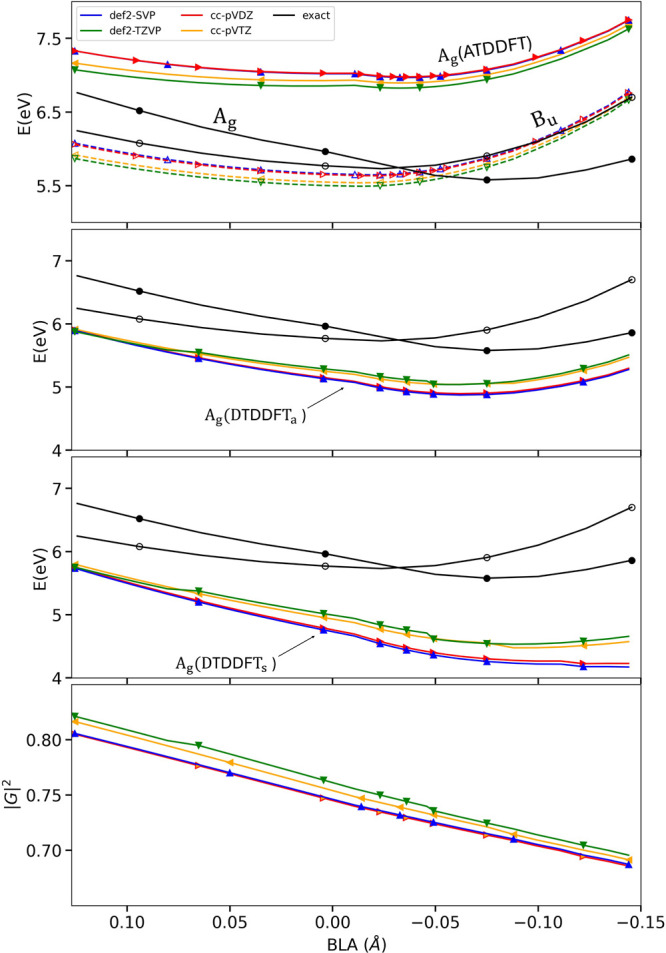
This is the corrected version of Figure 2 of the original paper, showing basis-set convergence in DTDDFT for the energies as well as the values of |G|^2^.

**S1 fig3:**
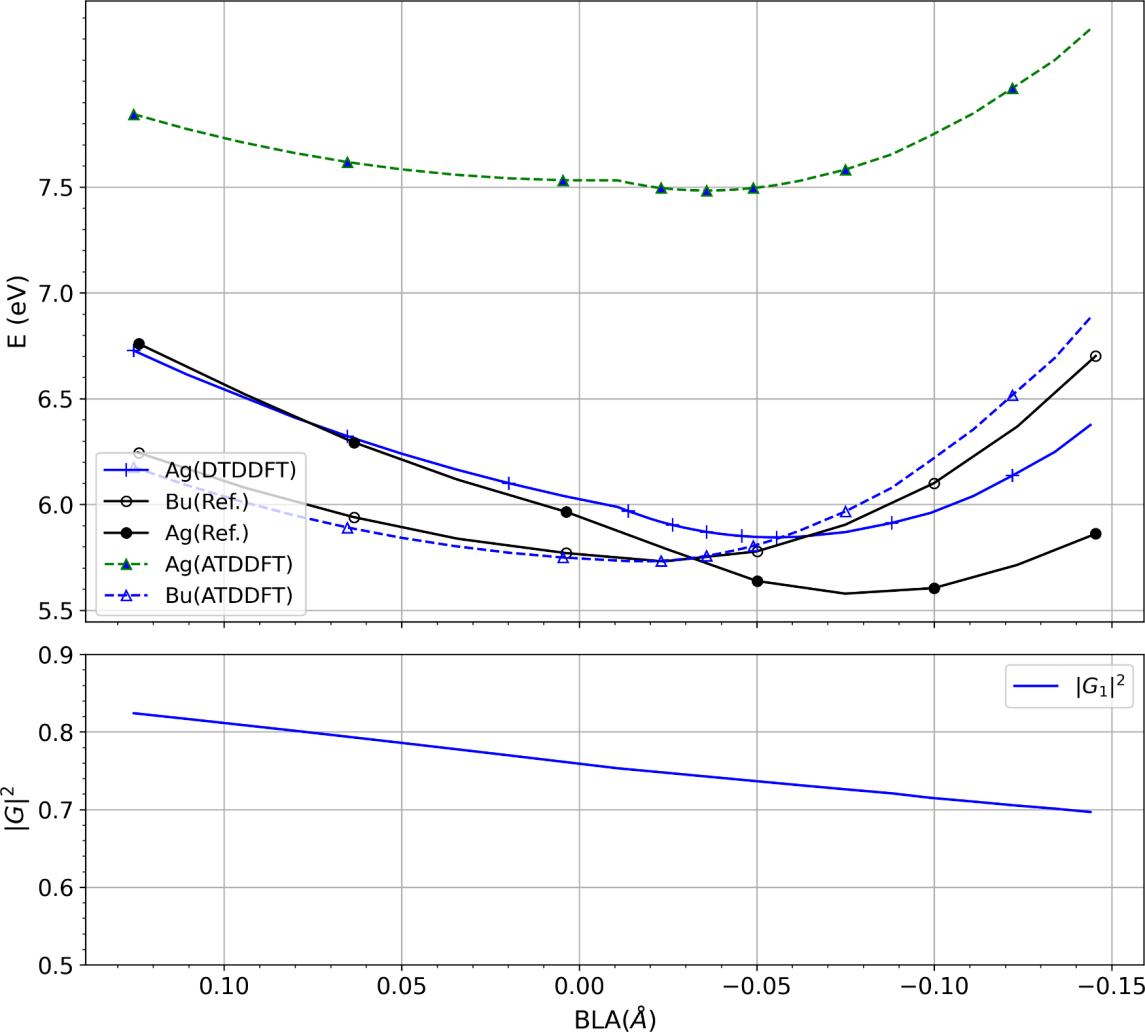
(Corresponding to panel 3 of Figure 1 of the Supporting Information) DTDDFT_a_, calculated according to the equations in the main paper, using the CAM-B3LYP functional and the def2-SVP basis.

When the calculations are performed according to the formulas presented in eqs 16, 17, 18, and 19, the results are generally less accurate than those reported in the original paper. Figure 1 below shows the corrected results for DTDDFT_*a*_ applied to PBE0/cc-pVTZ and should replace Figure 1 of the original paper, while Figure 3 presents the same for CAM-B3LYP/def2-SVP and should replace the lowest panel of Figure 1 of the Supporting Information. Notably, the latter remains quite accurate, closely following the reference A_*g*_ state curve and capturing the curve-crossing at a bond-length alternation (BLA) close to the exact value. In contrast, when applied to PBE0, while DTDDFT does improve the slope and trend of the curve compared to ATDDFT, there is an underestimation that shifts the curve-crossing further from the reference.

Therefore, a conclusion of our paper that “the DTDDFT error for the state of double-excitation character Ag is similar to error that adiabatic TDDFT makes for the single-excitation Bu, when the DTDDFT is built from the same adiabatic xc functional”, is incorrect; the error is certainly much less than it is for adiabatic TDDFT and the state character is more accurately captured, but depending on the functional, the error may be larger than the error that functional makes for the singly excited states. The conclusions we had originally made may be true for the original derived formulas of DTDDFT applied on top of some adiabatic functionals (e.g., CAM-B3LYP). If applied on top of the main adiabatic functional studied in the original paper, PBE0, the results, while generally improving over adiabatic PBE0 in trends over the BLA range shown, are not so accurate and yield a curve-crossing somewhat displaced from that predicted by the wave function reference. When applied on top of PBE, the results are also not accurate, as observed in our original paper. This could be because of charge-transfer character in the Bu excitation, which enters into the dressing matrix elements for the Ag state.

The general framework of the DTDDFT approach is still valid, correcting the character of states of double-excitation character, and giving improved energies compared with the adiabatic approximation. Further investigation is needed as to whether the modified DTDDFT approach with the factor of 1-half and modified dressing elements can be justified in certain cases, given the increased accuracy for a wider range of base adiabatic functionals.

