# Supplementary material for: Capturing the Elusive Curve-Crossing in Low-Lying States of Butadiene with Dressed TDDFT
Source: J Phys Chem Lett. 2025 Jan 10;16(3):703–9. doi: 10.1021/acs.jpclett.4c03167 (PMC12333331; doi:10.1021/acs.jpclett.4c03167)
Supplement: Supplementary file 1 [file jz4c03167_si_001.pdf]

Supplementary Material for ”Capturing the elusive curve-crossing in low-lying states of butadiene with Dressed TDDFT”

Davood Dar[1] and Neepa T. Maitra[1]

[1]*Department of Physics, Rutgers University, Newark 07102, New Jersey USA*

(Dated: January 7, 2025)

I. REDUCTION OF DTDDFT KERNEL TO DTDA

Here we show that the dressed frequency-dependent kernels derived in the main text reduce to the DTDA expression from references[1–3] when backward transitions are neglected, as in the Tamm-Dancoff approach. We start with the dressing in Eq. (18) of the main text but keeping the term, $H_{qD}H_{Dq'}$ in the denominator from Eq. (10):

$$X_{qq'}(\omega) = \frac{H_{qD}H_{Dq'}}{4\sqrt{\nu_q\nu_{q'}}}\left[1 + \frac{(\nu_q + \nu_D)(\nu_{q'} + \nu_D)}{\omega^2 - (\nu_D^2 + H_{qD}H_{Dq'})}\right]. \tag{S.1}$$

Note that the analysis below proceeds similarly for the different variants. The Tamm-Dancoff approximation involves ignoring the backward transitions, i.e. by considering positive-frequencies far from negative-frequency roots. Let us expand around the positive root of the denominator, ω_+, where

$$\omega_+ = \sqrt{\nu_D^2 + H_{qD}H_{Dq'}}. \tag{S.2}$$

Near this root, we express ω as $\omega_+ + \delta$, where δ is a small deviation:

$$\omega = \omega_+ + \delta. \tag{S.3}$$

Expanding the denominator in terms of δ gives, up to order δ:

$$(\omega_+ + \delta)^2 - (\nu_D^2 + H_{qD}H_{Dq'}) = 2\omega_+\delta\,, \tag{S.4}$$

so that

$$\frac{1}{\omega^2 - (\nu_D^2 + H_{qD}H_{Dq'})} = \frac{1/2\omega_+}{\omega - \omega_+}. \tag{S.5}$$

Assuming the single KS excitation frequencies, ν_q and $\nu_{q'}$ lie close to each other and also to the double excitation frequency ν_D, the above dressing reduces to

$$X_{qq'}(\omega) = \frac{H_{qD}H_{Dq'}}{4\nu_D}\left[1 + \frac{2\nu_D^2/\omega_+}{(\omega - \omega_+)}\right] \tag{S.6}$$

Near a double excitation, the last term in the brackets becomes much larger than 1, so we can write the above equation as

$$X_{qq'}(\omega) = \frac{H_{qD}H_{Dq'}(\nu_D/\sqrt{\nu_D^2 + H_{qD}H_{Dq'}})}{2\left(\omega - \sqrt{\nu_D^2 + H_{qD}H_{Dq'}}\right)}. \tag{S.7}$$

Recalling that $X_{qq'}$ is added to the adiabatic part of the xc kernel, we now compare this equation with the kernel given in Ref. [3], which was derived from the original dressed Tamm-Dancoff approach, and using the analog of what we call here the $\mathrm{DTDDFT_S}$ variant. The result from Ref. [3] is

$$f^{\mathrm{DTDA}}_{\mathrm{XC}\,qq'}(\omega) = f^{\mathrm{adia}}_{\mathrm{XC}\,qq'} + \frac{H_{qD}H_{Dq'}}{2(\omega - \nu_D)}, \tag{S.8}$$

from which we see that the dressing term (second term in the above equation) agrees with ours once we replace $\nu_D^2 + H_{qD}H_{Dq'}$ with ν_D^2. As discussed in the main text of the paper, having $H_{qD}H_{Dq'}$ in the denominator of the dressing makes it ill-defined by giving it an arbitrary sign-dependence. Here we see another reason to neglect it: ignoring this term makes our kernel agree with the one given in Ref. [1–3] derived from the dressed Tamm-Dancoff approach.

II. RESULTS FOR PBE, PBE0 AND CAM-B3LYP

Here we investigate the performance of $\mathrm{DTDDFT_a}$ for the butadiene curve-crossing when it is built upon three different types of adiabatic functionals: a GGA (PBE), the hybrid PBE0 that was presented in the main text, and a range-separated hybrid CAM-B3LYP. The figure shows the results computed in the def2-SVP basis. We see that

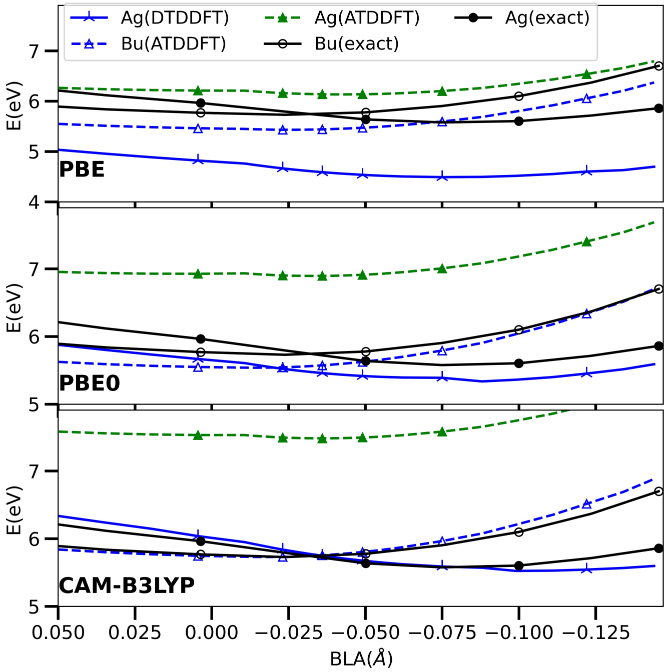

FIG. 1. Comparison of $\mathrm{DTDDFT_a}$ energies based on PBE, PBE0, and CAM-B3LYP for the adiabatic functional energies (top, middle, and lowest, respectively). As in Fig 1 of the main paper, the respective ATDDFT energies for 1Bu and 2Ag are shown, along with the reference 'exact' from δ-CR-EOMCC(2,3).

while predictions from all three functionals are similar for the 1Bu state, with the most accurate being CAM-B3LYP, their $\mathrm{DTDDFT_a}$ renditions differ more for the 2Ag state, again with CAM-B3LYP being most accurate. Although the trend of the exact curve is well-captured (unlike adiabatic PBE), $\mathrm{DTDDFT_a}$ with PBE is a significant underestimate of about 1eV, too low to display a crossing in the right region. This underestimate can be seen to be largely inherited from the underestimate of the adiabatic energy rather than the dressing per se. On the other hand the CAM-B3LYP is remarkably accurate. However, in our evaluation of CAM-B3LYP, we observed that while it provided accurate results, its performance displayed a notable sensitivity to the basis set. Specifically, results in the def2-TZVP (or cc-pVTZ) basis were slightly less accurate than those in the def2-SVP (or cc-pVDZ) basis. This highlights dependence on the basis set for this range-separated hybrid functional in the context of $\mathrm{DTDDFT_a}$. Addressing the origin of this sensitivity lies beyond the scope of the present study but is an important consideration for the broader applicability of the method.

[1] R. J. Cave, F. Zhang, N. T. Maitra, and K. Burke, Chem. Phys. Lett. **389**, 39 (2004).
[2] N. T. Maitra, F. Zhang, R. J. Cave, and K. Burke, J. Chem. Phys. **120** (2004).
[3] G. Mazur and R. Włodarczyk, J. Comput. Chem. **30**, 811 (2009).
